# Blood flow restriction as a potential therapy to restore physical function following COVID-19 infection

**DOI:** 10.3389/fphys.2023.1235172

**Published:** 2023-07-21

**Authors:** Isaac J. Wedig, John J. Durocher, John McDaniel, Steven J. Elmer

**Affiliations:** ^1^ Department of Kinesiology and Integrative Physiology, Michigan Technological University, Houghton, MI, United States; ^2^ Health Research Institute, Michigan Technological University, Houghton, MI, United States; ^3^ Department of Biological Sciences and Integrative Physiology and Health Sciences Center, Purdue University Northwest, Hammond, IN, United States; ^4^ Department of Exercise Physiology, Kent State University, Kent, OH, United States

**Keywords:** post-acute sequelae of SARS-CoV-2, occlusion training, muscle strength, aerobic capacity, pandemic, pathophysiology

## Abstract

Accumulating evidence indicates that some COVID-19 survivors display reduced muscle mass, muscle strength, and aerobic capacity, which contribute to impairments in physical function that can persist for months after the acute phase of illness. Accordingly, strategies to restore muscle mass, muscle strength, and aerobic capacity following infection are critical to mitigate the long-term consequences of COVID-19. Blood flow restriction (BFR), which involves the application of mechanical compression to the limbs, presents a promising therapy that could be utilized throughout different phases of COVID-19 illness. Specifically, we hypothesize that: 1) use of passive BFR modalities can mitigate losses of muscle mass and muscle strength that occur during acute infection and 2) exercise with BFR can serve as an effective alternative to high-intensity exercise without BFR for regaining muscle mass, muscle strength, and aerobic capacity during convalescence. The various applications of BFR may also serve as a targeted therapy to address the underlying pathophysiology of COVID-19 and provide benefits to the musculoskeletal system as well as other organ systems affected by the disease. Consequently, we present a theoretical framework with which BFR could be implemented throughout the progression from acute illness to outpatient rehabilitation with the goal of improving short- and long-term outcomes in COVID-19 survivors. We envision that this paper will encourage discussion and consideration among researchers and clinicians of the potential therapeutic benefits of BFR to treat not only COVID-19 but similar pathologies and cases of acute critical illness.

## Introduction

To date, there have been over 759 million reported cases of coronavirus disease 2019 (COVID-19) and over 6.8 million deaths worldwide (World health Organization, 2023). In addition to the acute complications associated with COVID-19 infection, accumulating evidence (Groff et al., 2021; Huang et al., 2021; Lopez-Leon et al., 2021; Nasserie et al., 2021; Salamanna et al., 2021) indicates that a variety of symptoms can persist for weeks and/or months following the acute phase of illness (i.e., long COVID, post-acute sequelae of COVID-19, post-COVID-19 syndrome). Among the most prevalent symptoms are fatigue, dyspnea, cognitive dysfunction, muscle and joint pain, and weakness. Moreover, physical function, which is the ability to move around and perform daily activities, can be impaired for up to 6 months following acute illness (de Oliveira Almeida et al., 2022). While these outcomes have been reported across acute illness severities, individuals with more severe illness requiring hospitalization appear to be most affected.

Physical function is influenced by the integration of multiple organ systems, particularly the musculoskeletal and cardiorespiratory systems. Accordingly, skeletal muscle mass and muscular strength (Wang et al., 2020), as well as aerobic capacity (Misic et al., 2007) (i.e., peak oxygen consumption), are important determinants of physical function. Individuals who become critically ill with COVID-19 experience rapid muscle wasting (de Andrade-Junior et al., 2021), loss of muscle strength (de Andrade-Junior et al., 2021; Paneroni et al., 2021), and reduced aerobic capacity (Baratto et al., 2021) during hospitalization. Furthermore, these losses are not recovered months following acute infection. Ramirez-Velez and colleagues (Ramírez-Vélez et al., 2022) reported low muscle mass and strength in COVID-19 survivors at 3 months following acute illness. Aparisi and colleagues (Aparisi et al., 2022) also reported lower aerobic capacity in survivors with some evidence indicating that impairments may persist up to 12 months after initial infection. Together, these data suggest that diminished skeletal muscle mass, muscle strength, and aerobic capacity are likely contributors to long-term impairments in physical function. The mechanisms responsible for these effects are not well understood (Ferreira and Oliveira, 2021; Seixas et al., 2022; Serviente et al., 2022) and may be multifactorial including factors associated with general critical illness (i.e., extended periods of inactivity, pharmacological therapies, malnutrition) and/or mechanisms specific to COVID-19 pathophysiology (i.e., direct viral infiltration, renin angiotensin system dysregulation, systemic inflammation, and oxidative stress).

Collectively, the chronic manifestations of COVID-19 infection may be comprising long-term health and setting those individuals who become infected on a path toward frailty and disease. Persistent physical function impairments following COVID-19 occur in both middle aged and older adults and are associated with lower physical activity levels (Delbressine et al., 2021; Ramírez-Vélez et al., 2022), increased risk of sarcopenia (Xu et al., 2022), and may increase chronic conditions such as obesity, cardiovascular disease, and diabetes. Furthermore, long-term physical functional impairments may drastically impact the workforce. A recent report (Ladlow et al., 2023) indicated that half of British Armed Forces were medically non-deployable at 12 months after COVID-19 infection. As COVID-19 continues to impact the world, the health and economic consequences of long-term symptoms could be astronomical.

Currently, evidence-based strategies for restoring physical function in those individuals suffering from short-to long-term complications following COVID-19 are limited. Developing safe, feasible, and cost-effective interventions to mitigate the loss of muscle mass, muscle strength, and aerobic capacity are of paramount importance and align with COVID-19 initiatives (US Department of Health and Human Services, 2022). Based on the unique symptoms, pathophysiology, and challenges associated with COVID-19, innovative rehabilitation strategies are required. Recently, Udina and colleagues (Udina et al., 2021) demonstrated that a multicomponent exercise intervention consisting of aerobic, resistance, and balance exercise (30 min/day, 7 days/wk) resulted in improved physical function in COVID-19 patients. Some individuals with COVID-19, however, may not be able to perform or tolerate such an aggressive exercise regimen that includes movements performed at moderate- and high-intensity. Alternatively, blood flow restriction (BFR), a modality for increasing muscle mass, muscle strength, and aerobic capacity while training at relatively low-intensity, may have application following COVID-19. Indeed, some authors have suggested the use of BFR as an intervention to counteract muscle and strength loss during the COVID-19 pandemic (de Oliveira et al., 2022) and as a treatment strategy for COVID-19 patients (Roman-Belmonte et al., 2020). Accordingly, the present paper aims to discuss the potential use of blood flow restriction (BFR) as a rehabilitation modality during and following COVID-19 infection to improve physical function.

## Hypothesis

Our working hypothesis is that implementation of BFR can facilitate recovery of physical function following COVID-19 infection. Specifically, we hypothesize that BFR can be applied during: 1) acute infection in those individuals with critical illness to mitigate the loss of muscle mass and muscle strength and 2) convalescence in those individuals recovering from critical illness to regain muscle mass, muscle strength, and aerobic capacity. To support these hypotheses, we first describe how BFR has been used with a broad range of populations and subsequently provide a rationale for how BFR offers a targeted therapy that specifically addresses the underlying pathophysiology of COVID-19. We also present a theoretical framework for using BFR throughout the progression from acute illness to outpatient rehabilitation.

## Blood flow restriction

**FIGURE 1:**
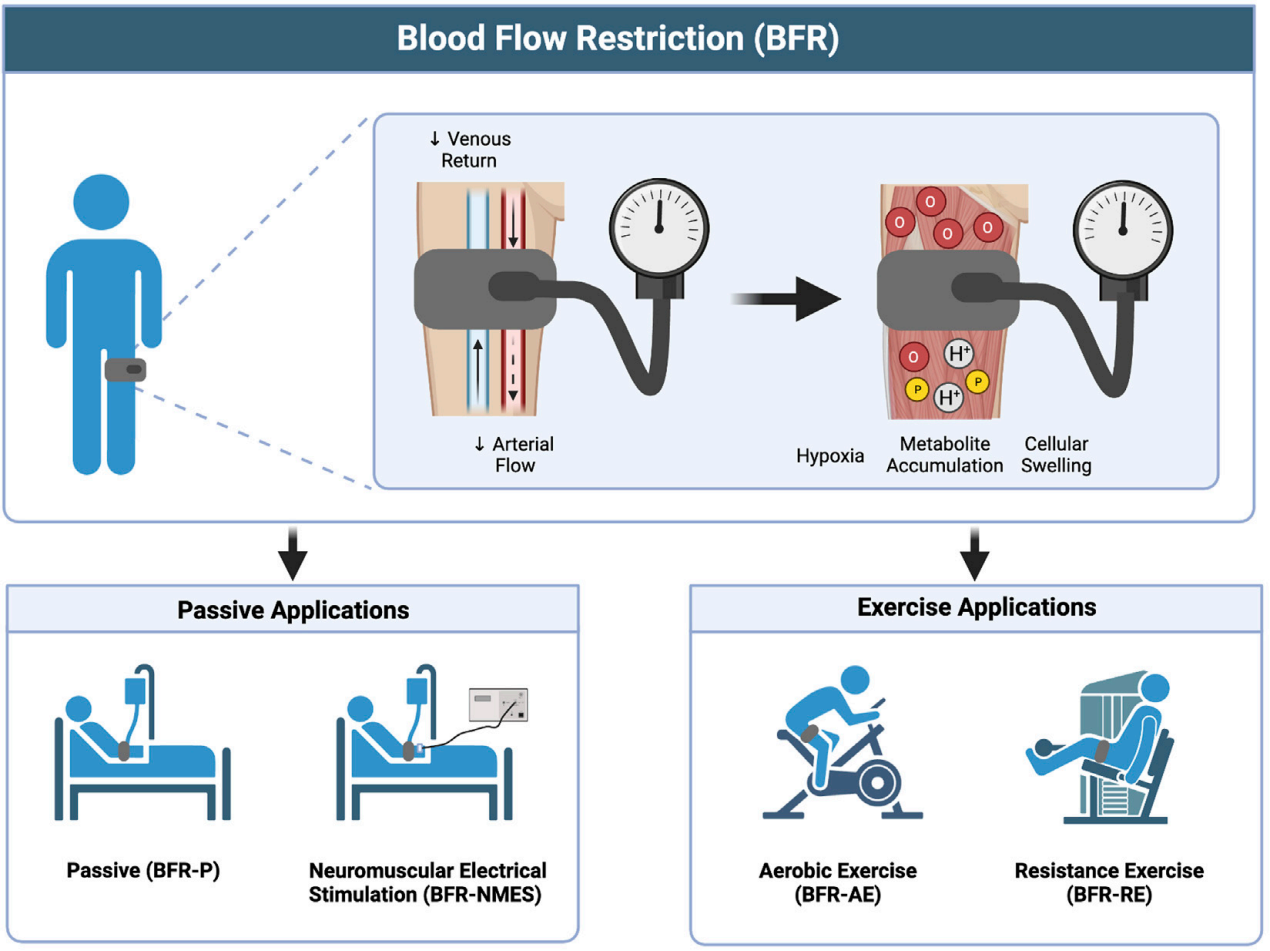
Overview of BFR and the different methods of application. Image created with BioRender and published with permission.

To date, there are more than 50 reviews published in applied physiology, exercise and sport science, and rehabilitation journals that discuss the application, effectiveness, and safety of BFR with populations ranging from adults living with chronic disease to elite athletes. Briefly, this modality (Figure 1) involves applying mechanical compression to the proximal portion of a limb, typically with a pneumatic cuff, which serves to partially reduce arterial blood flow to the limb while limiting most of the venous return (Kilgas et al., 2019). The reduced blood flow causes localized tissue hypoxia (Ilett et al., 2019), metabolite accumulation, and cellular swelling (Loenneke et al., 2012a) which may help to augment changes in muscle size, muscle strength, and/or aerobic capacity. Blood flow restriction is endorsed by the American Physical Therapy Association and is used in rehabilitation. It has been implemented with a variety of clinical populations including individuals with advanced age (Bennett and Slattery, 2019; Centner et al., 2019; Gronlund et al., 2020), orthopedic limitations (Hughes et al., 2017), critical illness (Barbalho et al., 2019), cardiovascular disease (Nakajima et al., 2010; Madarame et al., 2013; Kambič et al., 2019; Ogawa et al., 2021), hypertension (Wong et al., 2018), diabetes (Fini et al., 2021; Malekyian Fini et al., 2021), renal dysfunction (Corrêa et al., 2021a; Corrêa et al., 2021b), and neurological conditions (Gorgey et al., 2016; Yasuda et al., 2021; Douris et al., 2022). Notably, some of these conditions share similar pathophysiological presentations to COVID-19, characterized by increased levels of inflammation, oxidative stress, autonomic, and endothelial dysfunction.

Most commonly, BFR has been applied in combination with the performance of voluntary exercise, including both resistance exercise (BFR-RE) (Loenneke et al., 2012b; Slysz et al., 2016; Grønfeldt et al., 2020) and aerobic exercise (BFR-AE) (Bennett and Slattery, 2019; Formiga et al., 2020). Additionally, it has been implemented passively in the absence of muscle contraction (BFR-P) (Barbalho et al., 2019; Cerqueira et al., 2020) and in combination with involuntary muscle contraction elicited via neuromuscular electrical stimulation (BFR-NMES) (Natsume et al., 2015; Gorgey et al., 2016; Slysz and Burr, 2018). These applications of BFR may have use during the different phases of acute infection and post-acute recovery from COVID-19. Specifically, we propose that passive applications of BFR (BFR-P and BFR-NMES) can help to mitigate losses in muscle mass and muscle strength during acute COVID-19 illness and that the combination of BFR with exercise (BFR-AE and BFR-RE) can provide a viable way to restore muscle mass, muscle strength, and aerobic capacity to adequate levels during convalescence.

### Hypothesis 1—mitigate muscle and strength loss during acute infection

Muscle and strength loss are common during admittance to the intensive care unit (ICU) (Schefold et al., 2020) and correlate with hospital length of stay (Gruther et al., 2008) and physical function after discharge (Mayer et al., 2020). de Andrade-Junior and colleagues (de Andrade-Junior et al., 2021) reported that after 10 days in the ICU, COVID-19 patients displayed a 30% reduction in rectus femoris muscle cross-sectional area and a 19% reduction in the thickness of the anterior compartment of the quadriceps muscles. These rates of muscle loss are greater than those reported in other critically ill patients during ICU admission (Puthucheary et al., 2013). At hospital discharge, Paneroni and colleagues (Paneroni et al., 2021) reported that 80% of COVID-19 patients presented with muscle weakness and displayed quadriceps and biceps brachii muscle strength that were 54% and 69% of predicted values. Furthermore, accumulating evidence (Piotrowicz et al., 2021) indicates that COVID-19 survivors are at an increased risk of developing acute sarcopenia. Efforts to reduce rates of muscle and strength loss during severe acute COVID-19 infection may improve patient outcomes and reduce the time needed to recover physical function to adequate levels following discharge. However, viable therapies to mitigate the effects of critical illness on skeletal muscle are limited as hospitalized patients typically experience prolonged immobility and have a reduced ability to perform voluntary muscle contractions. As described below, the application of BFR-P and BFR-NMES may help to slow the rate of muscle and strength loss in those individuals hospitalized with severe COVID-19 illness.

#### BFR-P

Emerging evidence (Barbalho et al., 2019; Cerqueira et al., 2020) indicates that the intermittent application of BFR passively in the absence of muscle contraction mitigates losses in muscle and strength that occur during immobilization. Barbalho and colleagues (Barbalho et al., 2019) demonstrated that the addition of BFR to passive mobilization reduced rates of muscle wasting in older adults admitted to the ICU with coma. Compared to a control limb receiving passive mobilization alone, the addition of a tourniquet cuff to the proximal thigh during once daily passive mobilization decreased the rate of quadriceps muscle loss by 6% over an 11 day period. Other reports, which have been previously reviewed (Cerqueira et al., 2020), indicate that a BFR-P protocol consisting of 5 sets of 5 min restriction and 3 min reperfusion performed twice daily diminished disuse of the knee extensors by 11% following anterior crucial ligament reconstruction (Takarada et al., 2000) and prevented strength losses during 2 weeks of simulated cast immobilization in healthy adults (Kubota et al., 2008; Kubota et al., 2011). Although the mechanisms underlying these effects are largely unknown, it has been hypothesized (Loenneke et al., 2012a) that cellular swelling as a result of venous pooling may enhance muscle retention by inhibiting protein breakdown and/or increasing protein synthesis.

#### BFR-NMES

Neuromuscular electrical stimulation (NMES) is a technique that consists of generating involuntary muscle contractions using low level electrical currents delivered through electrodes applied to the skin. The addition of NMES to standard care (Liu et al., 2020) in critically ill patients reduces the rate of muscle loss, improves muscle strength, shortens length of stay in the hospital, and improves ability to perform activities of daily living. Some evidence (Natsume et al., 2015; Gorgey et al., 2016; Slysz and Burr, 2018) indicates that low-intensity NMES combined with BFR promotes more robust effects on muscle size and strength than low-intensity NMES or BFR-P performed alone. For example, Gorgey and colleagues (Gorgey et al., 2021) reported that 6 weeks of BFR-NMES in individuals living with spinal cord injury increased wrist extensor muscle cross-sectional area and improved electronically evoked wrist extensor torque. Changes in wrist extensor cross-sectional area were 17% greater in the treatment limb receiving BFR-NMES compared to a control limb receiving NMES alone. In another report (Natsume et al., 2015), BFR-NMES performed twice daily (5 days/week) in the lower-body increased quadriceps muscle thickness and maximal knee extension strength after 2 weeks of training in young males. No changes were observed in a control limb performing NMES alone which is consistent with related reports (Slysz and Burr, 2018).

#### Pathophysiology of COVID-19

Endothelial dysfunction has been suggested to be a major pathogenic mechanism of COVID-19 (Del Turco et al., 2020; Bonaventura et al., 2021) and persists for months beyond acute infection (Serviente et al., 2022). Endothelial dysfunction is associated with numerous chronic diseases (Hadi et al., 2005) as well as risk of future cardiovascular events (Green et al., 2011) and likely contributes to long-term symptoms in COVID-19 survivors (Charfeddine et al., 2021). In a systematic review and meta-analysis including 292 participants, Gu and colleagues (Gu et al., 2021) reported that BFR-P protocols, referred to as ischemic preconditioning, augment endothelial function via increased flow mediated dilation. Several authors (Jeffries et al., 2018; Rytter et al., 2020) have also reported enhanced microvascular function when implementing similar protocols. Like BFR-P protocols discussed previously, ischemic preconditioning involves the cyclical application of blood flow restriction and reperfusion, however, tourniquets are applied at higher pressures that result in complete arterial occlusion. A large body of evidence (Stokfisz et al., 2017) demonstrates that ischemic preconditioning protects tissues from subsequent ischemia and reperfusion injury and that these effects also occur in remote tissues (i.e., remote ischemic conditioning) that are not directly subject to the localized ischemic preconditioning stimulus. Indeed, lung and cardiovascular injury (Guzik et al., 2020) are common with severe COVID-19 illness and ischemic preconditioning may confer a systemic protective effect. The use of ischemic preconditioning in COVID-19 patients has been previously suggested (Incognito et al., 2021; Cahalin et al., 2023). Additionally, COVID-19 patients display impaired hemostasis (Hanff et al., 2020) which is characterized by overactivation in the coagulation system with reduced fibrinolytic activity. Accordingly, thrombotic complications are common in COVID-19. Longstanding evidence indicates that vascular compression stimulates the fibrinolytic system without elevating the coagulation cascade (Holemans, 1963; Robertson et al., 1972; Stegnar and Pentek, 1993; Kohro et al., 2005). Accordingly, when applied in COVID-19 patients, various BFR-P approaches could potentially help to reduce risk for thrombotic complications. While there is extensive literature supporting the application of BFR-P and its effects on numerous organ systems, reports implementing BFR-NMES are limited. To the best of our knowledge, only one report has investigated the effects of BFR-NMES on vascular function in which the authors (Gorgey et al., 2016) demonstrated acute increases in brachial artery flow mediated dilation following BFR-NMES when compared BFR alone. These preliminary data suggest vascular benefits with the addition of NMES, however, more work is needed to characterize the effects of BFR-NMES.

**FIGURE 2:**
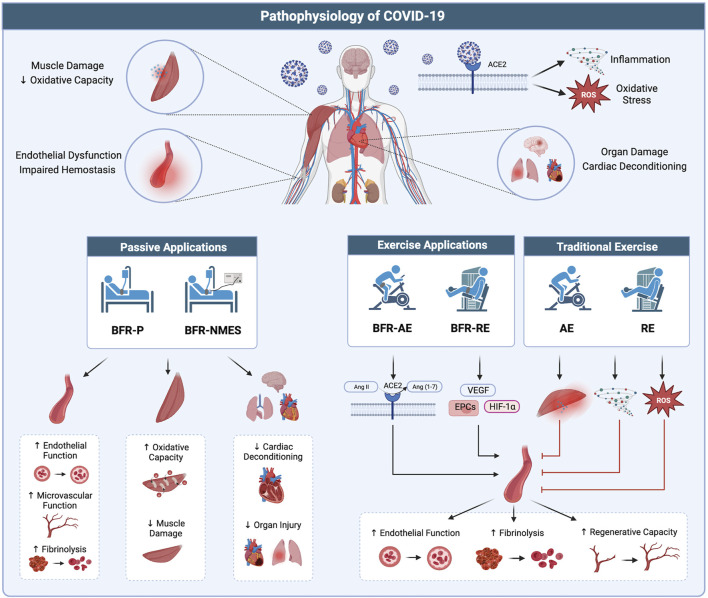
Potential therapeutic benefits of BFR in treating the pathophysiology of COVID-19. (Top) Infection with COVID-19 results in widespread organ dysfunction which may be the result of systemic viral infiltration, hyper-inflammation, and oxidative stress. (Bottom left) Passive applications of BFR (BFR-P and BFR-NMES) promote positive effects in the vasculature, skeletal muscle, and vital organs which may serve to combat multiple organ dysfunction occurring with COVID-19. (Bottom right) Exercise applications of BFR (BFR-AE and BFR-RE) promote benefits to the vascular system through increased ACE2 activity, stimulating the release of hematopoietic stem cells, and promoting the expression of factors related to vascular growth and regeneration. Additionally, compared to high-intensity exercise without BFR, low-intensity exercise with BFR results in lower levels of muscle damage, inflammation, and oxidative stress, which could exacerbate the pathophysiological mechanisms of COVD-19 and worsen symptoms. Image created with BioRender and published with permission.

Low aerobic capacity in COVID-19 survivors, as assessed through an incremental exercise test for determination of VO_2peak_, has been attributed to both central and peripheral factors (Aparisi et al., 2022). Thus, impairments throughout the oxygen transport pathway are likely present. In addition to potentially enhancing oxygen delivery via improved peripheral vascular function, BFR-P could attenuate reductions in aerobic capacity during critical COVID-19 illness by reducing cardiac deconditioning and improving oxygen kinetics in skeletal muscle. Nakajima and colleagues (Nakajima et al., 2008) reported similar hemodynamic responses to that of upright standing when BFR-P was applied to the proximal thighs of participants placed in a 6-degrees head-down tilt position. These data let us speculate that BFR-P could approximate the cardiac demands of standing and attenuate cardiac deconditioning and orthostatic intolerance occurring during prolonged bedrest. Additionally, some authors (Saito et al., 2004; Patterson et al., 2015) have reported that repeated ischemic preconditioning exposure improves local skeletal muscle oxygen dynamics during exercise. Data from Jeffries and colleagues (Jeffries et al., 2018) demonstrated that 7 consecutive days of lower-body ischemic preconditioning increased local skeletal muscle oxidative capacity. Together, BFR-P protocols could help to preserve skeletal muscle mass and strength during critical illness and offer a systemic strategy that can provide benefits to the musculoskeletal system and possibly other organ systems, some of which are affected during COVID-19 infection (Figure 2; bottom left).

### Hypothesis 2—increase muscle mass, muscle strength, and aerobic capacity during convalescence

Exercise training is a promising therapy in the rehabilitation of COVID-19 as it: 1) promotes healthy function in multiple organ systems, 2) effectively treats a variety of diseases that share similar pathophysiological presentations to COVID-19, 3) increases muscle mass, muscle strength, and aerobic capacity, and 4) improves physical function. A recent systematic review (Ahmadi Hekmatikar et al., 2022) including 233 COVID-19 survivors found that a combination of aerobic and resistance exercise training following hospital discharge increased muscle strength, physical function, and quality of life. It is important to note that several concerns have been raised about exercise after COVID-19 including the risk of cardiac injury, thromboembolic complications, and post-exertional symptom exacerbation (Salman et al., 2021; World Physiotherapy, 2021). Given these concerns, along with frequently reported symptoms of fatigue, joint and muscle pain, and weakness, exercise prescription in COVID-19 survivors requires careful consideration. Indeed, higher exercise intensities needed to promote increases in muscle size, strength, and aerobic capacity may be challenging or contraindicated. Alternatively, exercise training with BFR could offer a unique approach for COVID-19 survivors to attain the benefits of high-intensity exercise. The main advantages of exercise with BFR compared to traditional exercise are: 1) increases in muscle size, strength, and aerobic capacity can be achieved with lower exercise intensities (Loenneke et al., 2012b; Bennett and Slattery, 2019; Clarkson et al., 2019), 2) adaptations from BFR occur faster, and 3) muscle size and strength can be increased with both aerobic and resistance exercise (Slysz et al., 2016). Although the exact mechanisms responsible for these adaptations are unknown, evidence (Jessee et al., 2018) suggests that increases in muscle size and strength are likely driven by cellular swelling and increased muscle activation occurring due to metabolite induced fatigue. Currently, increases in aerobic capacity are thought to occur via enhanced conduit artery blood flow, muscle capillary density, and muscle oxidative capacity in response to both the hypoxic stimulus during exercise and increased vascular shear stress upon cuff release (Formiga et al., 2020). A more comprehensive discussion surrounding the mechanisms responsible for adaptions to exercise with BFR are reviewed by Jessee and colleagues (Jessee et al., 2018) and Pignanelli and colleagues (Pignanelli et al., 2021). The following sections briefly discuss the effects of BFR-AE and BFR-RE on muscle size, muscle strength, and aerobic capacity and highlight unique advantages of these exercise modalities over that of high-intensity exercise without BFR in potentially managing the pathophysiology of COVID-19.

#### BFR-AE

The combination of aerobic exercise, such as walking or cycling, with BFR increases muscle size and strength in younger (Slysz et al., 2016) and older adults (Centner et al., 2019). Importantly, these adaptations are achieved at low exercise intensities (e.g., 45% heart rate reserve or 40% VO_2peak_) and occur as early as 3 weeks, sooner than that observed with high-intensity resistance training without BFR. In addition to increases in muscle size and strength, BFR-AE also facilitates increases in aerobic capacity in young adults (Bennett and Slattery, 2019; Formiga et al., 2020). Thus, BFR-AE provides an efficient exercise mode that improves both muscle size and strength as well as aerobic capacity simultaneously. Importantly, a systematic review by Clarkson and colleagues (Clarkson et al., 2019) indicated that adaptations to BFR-AE translate to improvements in objective measures of physical function, including the 30-s sit-to-stand, timed up and go, and 6-min walk test. This modality has been safely applied in individuals living with a variety of diseases including hypertension (Barili et al., 2018), end-stage kidney disease (Clarkson et al., 2020), chronic heart failure (Tanaka and Takarada, 2018), and obesity (Karabulut and Garcia, 2017).

#### BFR-RE

Increases in muscle size and strength with the performance of resistance exercise in combination with BFR have been reported in reviews of healthy young (Loenneke et al., 2012b; Slysz et al., 2016; Grønfeldt et al., 2020) and older populations (Centner et al., 2019; Grønfeldt et al., 2020), as well as those individuals with orthopedic limitations (Hughes et al., 2017). Adaptations from BFR-RE are achieved with lower exercise intensities (20%–40% 1RM) and are greater than those attained with low-intensity resistance exercise performed without BFR. Relative to BFR-AE, the magnitude of muscle size and strength improvements with BFR-RE are greater (Slysz et al., 2016) and also translate to improvements in objective measures of physical function (Clarkson et al., 2019; Baker et al., 2020). Few studies have investigated the effects of BFR-RE on aerobic capacity, however, one report (Nakajima et al., 2010) noted increases in aerobic capacity when BFR-RE was performed for 3 months in individuals living with ischemic heart disease. Thus, BFR-RE may have the potential to promote cardiovascular adaptations in diseased and less trained populations. This modality has been applied in individuals living with hypertension (Wong et al., 2018), diabetes (Fini et al., 2021; Malekyian Fini et al., 2021), chronic kidney disease (Corrêa et al., 2021a; Corrêa et al., 2021b), and cardiovascular disease (Nakajima et al., 2010; Fukuda et al., 2013; Madarame et al., 2013; Ishizaka et al., 2019; Kambič et al., 2019; Ogawa et al., 2021).

#### Pathophysiology of COVID-19

Elevated levels of inflammation and oxidative stress have been suggested (Del Turco et al., 2020) to play important roles contributing to organ dysfunction with COVID-19. Furthermore, evidence indicates that oxidative stress (Ratchford et al., 2021) and inflammation (Montefusco et al., 2021) remain elevated beyond acute infection and likely contribute to long-term symptoms. Accordingly, it is important that interventions aimed at restoring muscle mass, muscle strength, and aerobic capacity in COVID-19 survivors do not exacerbate the underlying pathological mechanisms of the disease. Traditional high-intensity exercise without BFR can result in acute elevations in oxidative stress, muscle damage, and inflammation (Cerqueira et al., 2019). These responses are greatest in individuals that are deconditioned and unaccustomed to exercise. Given the combination of prolonged immobilization, deconditioning, and pre-existing inflammatory and oxidant-antioxidant imbalances, the acute physiological perturbations associated with high-intensity exercise could be deleterious in those recovering from severe COVID-19. Additionally, meta-analyses (Li et al., 2020; Wu et al., 2020; Bansal et al., 2021) have reported elevated makers of skeletal muscle damage (i.e., creatine kinase, lactate dehydrogenase, myoglobin) associated with COVID-19 infection and case studies (Husain et al., 2020; Jin and Tong, 2020; Mukherjee et al., 2020) have documented rhabdomyolysis in patients. Exercise resulting in muscle damage and a subsequent inflammatory response could further deteriorate physical function, suppress the immune system, and worsen symptoms.

A recent systematic review and meta-analysis (Ferlito et al., 2023) indicates that low-intensity exercise with BFR results in lower acute elevations in biomarkers of oxidative stress when compared to high-intensity exercise without BFR. Additionally, Petrick and colleagues (Petrick et al., 2019) demonstrated that skeletal muscle mitochondrial reactive oxygen species emission rates were acutely decreased 2 h following low-intensity BFR-RE but not after the same exercise protocol performed without BFR. Evidence (Loenneke et al., 2014; Nielsen et al., 2017) also suggests that low-intensity BFR-RE results in minimal muscle damage based on direct (integrity of muscle fibers) and indirect (alterations in muscle strength, range of motion, blood markers) assessments. Accordingly, exercise with BFR provides a novel method to increase muscle size, muscle strength, and aerobic capacity which elicits relatively smaller acute elevations in oxidative stress and muscle damage compared to high-intensity exercise without BFR. Thus, this modality provides an alternative way to restore physical function that may be less likely to exacerbate pathophysiological mechanisms of COVID-19.

A potential mechanism by which COVID-19 promotes systemic pathology, particularly endothelial dysfunction, is interaction of SARS-CoV-2 with the renin angiotensin system (RAS). The principal target of SARS-CoV-2 binding is angiotensin-converting enzyme 2 (ACE2), a membrane bound protein found in numerous tissues throughout the body. The active form of ACE2 opposes the action of the RAS. Specifically, ACE2 degrades Angiotensin I (Ang I) and converts Angiotensin II (Ang II) into Ang (1,7), which exerts vasodilatory and anti-inflammatory effects. With COVID-19 infection, the consumption and downregulation of ACE2 via SARS-CoV-2 binding leaves RAS unopposed, increasing the ratio of ANG II to ANG (1,7) and drives excessive vasoconstriction, inflammation, and oxidative stress. Joshi and colleagues (Joshi et al., 2020) reported that BFR-RE performed in the lower-body substantially increased ACE2 activity and enhanced the ACE2-to-ACE ratio following exercise. Additionally, these authors reported increases in circulating hematopoietic stem/progenitor cells which were associated with three-fold increases in vascular endothelial growth factor receptors. Further, a recent meta-analysis (Li et al., 2022) demonstrated that exercise with BFR facilitates greater expression of angiogenesis related factors than exercise performed without BFR. Collectively, this evidence suggests that exercise with BFR may combat RAS dysregulation in COVID-19 and enhance the adaptive and regenerative capacity of the vascular system. Other data have reported direct benefits of exercise with BFR throughout the vascular tree. In a recent meta-analysis, Pereira-Neto and colleagues (Pereira-Neto et al., 2021) reported that 4 or more weeks of BFR-RE improves endothelial function (i.e., flow mediated dilation, reactive hyperemia blood flow, and reactive hyperemia index) and some data (Evans et al., 2010; Hunt et al., 2013) report enhanced capillary growth.

Among the benefits of exercise is its positive impact on hemostasis. High-intensity resistance training without BFR acutely enhances fibrinolytic activity (deJong et al., 2006), increasing tissue plasminogen activator (tPA) and decreasing plasminogen activator inhibitor-1 (PAI-1), without elevating activity in the coagulation system. Evidence indicates similar responses in the fibrinolytic system with the performance of low-intensity exercise with BFR. Nakajima and colleagues (Nakajima et al., 2007) reported significant increases in tPA antigen and unchanged PAI-1 activity during low-intensity BFR-RE (30% 1RM) performed after 24 h of bedrest. Similarly, Clark and colleagues (Clark et al., 2021) reported a 33% increase in tPA antigen immediately following acute bouts of BFR-RE with no alterations in markers of coagulation. Responses were similar to those observed with high-intensity resistance exercise without BFR. Furthermore, studies (Shimizu et al., 2016; Rapanut et al., 2019) implementing the chronic performance of BFR-RE have demonstrated decreases in von Willebrand factor (vWF) after 4 weeks. Taken together, these data demonstrate that exercise with BFR provides similar fibrinolytic effects as high-intensity exercise without BFR, albeit at lower exercise intensities, and could protect against short and long-term thrombotic complications associated with COVID-19. Exercise with BFR appears to promote a variety of positive adaptations in the vascular system and may confer several unique benefits to COVID-19 survivors that are not achieved with traditional higher intensity exercise (Figure 2; bottom right).

### Theoretical framework

**FIGURE 3:**
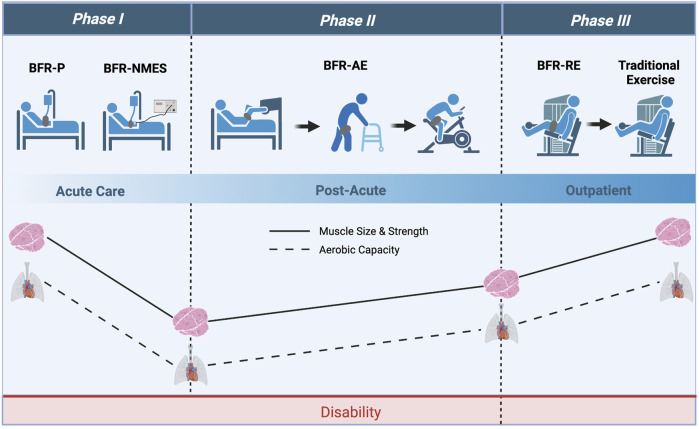
Theoretical framework with which BFR could be applied to COVID-19 survivors throughout acute care and outpatient rehabilitation. *Phase I* consists of using passive applications of BFR (BFR-P and BFR-NMES) to prevent losses in muscle mass and strength during acute care. *Phase II* consists of using various modes of BFR-AE to improve muscle mass, muscle strength, and aerobic capacity during post-acute care. Lastly, *Phase III* consists of using BFR-RE to provide further increases in muscle mass and strength while transitioning COVID-19 survivors to high-intensity exercise without BFR. Image created with BioRender and published with permission.

An evidence-based model of BFR progression from bed rest to outpatient rehabilitation for clinical populations was originally proposed by Loenneke et al. (2012c) and colleagues. The novelty of this approach is that BFR-assisted rehabilitation has the potential to reduce the time needed to reach the stage where patients can tolerate higher loads and intensities and thus accelerate recovery of physical function (Bielitzki et al., 2021). Here, we apply this model to COVID-19 and construct a theoretical framework for which BFR could be used with COVID-19 patients throughout the transition from acute illness to outpatient rehabilitation. As illustrated in Figure 3, our framework includes three phases of BFR application. ***Phase I*** consists of applying passive BFR applications (BFR-P and BFR-NMES) during severe acute COVID-19 illness to reduce muscle and strength loss while patients are immobilized. Importantly, these modalities can be implemented early in acute care and do not require active cooperation from the patient. Once capable of mobilization, patients can progress to ***Phase II***, which consists of performing BFR-AE to regain muscle mass, muscle strength, and aerobic capacity. Before patients are capable of ambulating, BFR-AE could be performed during early active mobilization activities such as bed mobility, transfers (e.g., supine-to-sit, sit-to-stand), arm ergometry, or supine leg ergometry. Once physically capable, patients can progress to more traditional BFR-AE exercise modes including walking and cycling. As patients’ mobility and tolerance to exercise increases, they can progress to ***Phase III***, which includes the addition of BFR-RE to provide a more robust method for increasing muscle mass and strength. Based on patient progress and physical ability, BFR-RE could be initiated in the post-acute rehabilitation setting or during outpatient rehabilitation. Given the substantial and prolonged decrements in aerobic capacity of COVID-19 survivors, it would be advised to continue BFR-AE during this phase and/or begin integrating high-intensity aerobic exercise without BFR based on patient tolerance. While initial resistance exercise training protocols can focus on BFR-RE exclusively, high-intensity resistance exercise without BFR should be slowly incorporated into the rehabilitation program as tolerated to stimulate additional improvements in muscle strength. Collectively, progression through each phase of BFR application can help to restore physical function and reduce the long-term consequences of severe COVID-19 infection. Notably, our framework is also consistent with other reports (de Oliveira et al., 2022) suggesting progressive application of BFR as a counteracting home-based intervention to maintain physical function during the COVID-19 pandemic.

**FIGURE 4:**
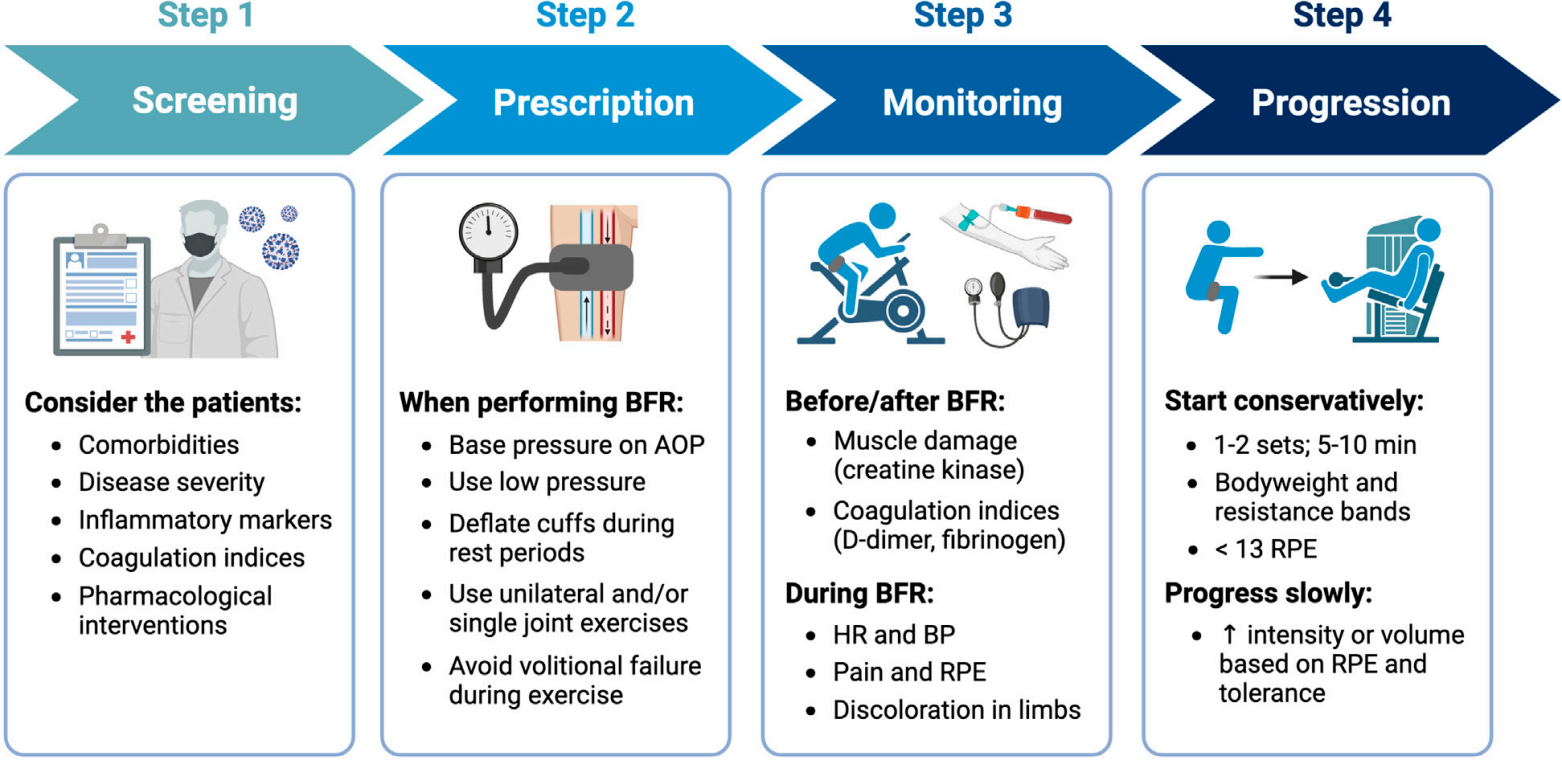
General recommendations for implementing BFR with COVID-19 survivors. Step 1 - screen patients for risk factors and/or contraindications, Step 2 - utilize prescriptions that minimize cardiovascular and perceptual demands, Step 3 - monitor patients before, during, and after performing BFR for adverse responses, and Step 4 - progress exercise slowly based on RPE and tolerance. Image created with BioRender and published with permission.

When implementing BFR with COVID-19 patients, there are several important factors to consider (Figure 4). First, robust screening for potential risk factors and/or contraindications is critical. Nascimento and colleagues (Nascimento et al., 2022) suggested that the decision to implement exercise with BFR in COVID-19 patients should consider each patient’s unique profile, including any pre-existing comorbidities, their disease severity, inflammatory markers, coagulation indices, and pharmacological interventions. Second, it is important to standardize the degree of blood flow restriction by basing cuff pressures on individual arterial occlusion pressure (McEwen et al., 2019), which is the minimum pressure required to occlude arterial blood flow to a limb. Moreover, acute cardiovascular and perceptual responses to exercise with BFR are reduced when utilizing lower cuff pressures (Mattocks et al., 2017), implementing intermittent *versus* continuous cuff pressure protocols (Brandner et al., 2015), selecting exercises that involve smaller amounts of muscle mass (Kilgas et al., 2019), and not performing exercise to volitional failure (Sieljacks et al., 2019). Third, initial BFR prescriptions for those individuals with or recovering from COVID-19 should be conservative and follow similar approaches to those used in other clinical populations. For example, when implementing BFR-RE in patients following cardiovascular surgery, Ogawa and colleagues (Ogawa et al., 2021) began with relatively low exercise intensities (e.g., 10%–20% 1RM) and volumes (1-2 exercises: 1 set x 20 repetitions). Fourth, hemodynamic (blood pressure, heart rate) and perceptual (perceived exertion, pain) responses should be carefully monitored during BFR and specific criteria (Nascimento et al., 2022) for stopping the modality should be followed. Additionally, markers of muscle damage (creatine kinase) and coagulation indices (D-dimer, fibrinogen) should also be monitored before and after exercise. Finally, once tolerance to BFR is established, intensity and volume can be slowly progressed based on the individual’s rate of perceived exertion during exercise. Guidelines for exercise progression in COVID-19 survivors based on perceived exertion have previously been recommended (Sari and Wijaya, 2023). It is important to note that some data (Clarkson et al., 2017) indicate that perceived exertion during exercise with BFR is highest during initial sessions but decreases with repeated exposure.

## Limitations and considerations

While BFR theoretically appears to be a viable solution for restoring physical function following COVID-19 infection, there are three notable limitations to our hypothesis. First is the safety of implementing BFR (Nascimento et al., 2022). Specifically, some authors (Spranger et al., 2015; Cristina-Oliveira et al., 2020) have appropriately raised concern for potential adverse cardiovascular responses to exercise with BFR in populations with cardiovascular disease (i.e., hypertension, heart failure, peripheral artery disease) who possess altered exercise pressor reflex function. The pathophysiology of COVID-19 resembles that of cardiovascular and inflammatory disease and those individuals developing severe COVID-19 illness are commonly older in age and have multiple pre-existing comorbidities. Furthermore, some evidence (Stute et al., 2022) indicates an augmented exercise pressor response in COVID-19 survivors. Therefore, concerns surrounding acute cardiovascular responses to exercise with BFR should be extended to those individuals infected with or recovering from COVID-19. Perhaps the biggest concern in this population is that of thrombotic complications given the high prevalence of hemostatic abnormalities. As stated above, robust screening for potential risk factors and/or contraindications is critical. Second, is the extent to which individuals could tolerate BFR. For example, low-intensity exercise with BFR generally leads to equal or only slightly lower ratings of perceived exertion and discomfort when compared to high-intensity exercise without BFR (De Queiros et al., 2022). Although exercise with BFR seems to be well tolerated in older adults and a variety of clinical populations, adoption and adherence may be challenging among those with and recovering from COVID-19 who display exercise intolerance. As discussed, modifications to various BFR prescriptions (i.e., cuff pressure, intermittent pressure application, exercise selection, and proximality to failure) may help to enhance exercise tolerance and adherence. Importantly, many COVID-19 survivors experience myalgic encephalomyelitits or chronic fatigue syndrome (ME/CFS) (Jason and Dorri, 2022) and may experience post-exertional malaise even with the performance of light exercise. Therefore, the utilization of any exercise type in COVID-19 survivors should exclude those individuals displaying symptoms consistent with ME/CFS. Lastly, is the capacity of medical professionals to implement BFR safely and effectively in clinical settings. Adequate training of BFR methodology and awareness of potential side effects and adverse outcomes is essential for making an informed decision about whether BFR is appropriate. Furthermore, access to proper technologies (i.e., cuffs and equipment for determining appropriate pressures) and knowledge of BFR exercise prescription plays a critical role in minimizing patient risk (Patterson et al., 2019). A comprehensive overview of BFR methodology, prescription, and safety is provided by Patterson and colleagues (Patterson et al., 2019).

## Summary

We hypothesize that the use of BFR could be an effective strategy to rehabilitate physical function in COVID-19 survivors. The application of BFR-P and BFR-NMES during acute infection has the potential to mitigate muscle and strength loss occurring with severe COVID-19 illness requiring hospitalization. During post-acute and outpatient rehabilitation, the combination of BFR with voluntary exercise (BFR-AE and BFR-RE) presents an alternative to high-intensity exercise without BFR to restore muscle mass, muscle strength, and aerobic capacity. Additionally, the various applications of BFR may offer a systemic therapy to combat organ dysfunction. A progressive model of BFR application throughout the phases of acute infection and rehabilitation offers a theoretical approach to address the long-term consequences of COVID-19. We hope that this paper encourages discussion and consideration among researchers and clinicians about the therapeutic potential of BFR to improve outcomes not only in COVID-19 survivors but in similar pathologies and cases of acute critical illness.

## Data Availability

The original contributions presented in the study are included in the article/Supplementary Material, further inquiries can be directed to the corresponding author.

## Abbreviations

ACE2, Angiotensin-Converting Enzyme 2; BFR, Blood Flow Restriction; BFR-RE, Resistance Exercise with Blood Flow Restriction; BFR-AE, Aerobic Exercise with Blood Flow Restriction; BFR-P, Passive Blood Flow Restriction; BFR-NMES, Neuromuscular Electrical Stimulation with Blood Flow Restriction; COVID-19, Coronavirus Disease 2019; ICU, Intensive Care Unit; VO2, Oxygen Consumption; 1RM, One-repetition Maximum; ME/CFS, Myalgic Encephalomyelitis or Chronic Fatigue Syndrome; PAI-1, plasminogen activator inhibitor-1; RAS, Renin Angiotensin System; tPA, Tissue Plasminogen Activator.
